# A Prompt Engineering Framework for Large Language Model–Based Mental Health Chatbots: Conceptual Framework

**DOI:** 10.2196/75078

**Published:** 2025-11-07

**Authors:** Sorio Boit, Rajvardhan Patil

**Affiliations:** 1 Department of Computer Science, College of Computing, Grand Valley State University, 1 Campus Dr, Allendale, MI, 49401, United States, 1 616-331-4375

**Keywords:** prompt engineering, ethical AI, conversational AI, mental health chatbot, AI in mental health care, digital mental health, large language model, artificial intelligence, MIND-SAFE framework

## Abstract

**Background:**

Artificial intelligence (AI), particularly large language models (LLMs), presents a significant opportunity to transform mental health care through scalable, on-demand support. While LLM-powered chatbots may help reduce barriers to care, their integration into clinical settings raises critical concerns regarding safety, reliability, and ethical oversight. A structured framework is needed to capture their benefits while addressing inherent risks. This paper introduces a conceptual model for prompt engineering, outlining core design principles for the responsible development of LLM-based mental health chatbots.

**Objective:**

This paper proposes the Mental Well-Being Through Dialogue – Safeguarded and Adaptive Framework for Ethics (MIND-SAFE), a comprehensive, layered framework for prompt engineering that integrates evidence-based therapeutic models, adaptive technology, and ethical safeguards. The objective is to propose and outline a practical foundation for developing AI-driven mental health interventions that are safe, effective, and clinically relevant.

**Methods:**

We outline a layered architecture for an LLM-based mental health chatbot. The design incorporates (1) an input layer with proactive risk detection; (2) a dialogue engine featuring a user state database for personalization and retrieval-augmented generation to ground responses in evidence-based therapies such as cognitive behavioral therapy, acceptance and commitment therapy, and dialectical behavior therapy; and (3) a multitiered safety system, including a postgeneration ethical filter and a continuous learning loop with therapist oversight.

**Results:**

The primary contribution is the framework itself, which systematically embeds clinical principles and ethical safeguards into system design. We also propose a comparative validation strategy to evaluate the framework’s added value against a baseline model. Its components are explicitly mapped to the Framework for AI Tool Assessment in Mental Health and Readiness Evaluation for AI-Mental Health Deployment and Implementation frameworks, ensuring alignment with current scholarly standards for responsible AI development.

**Conclusions:**

The framework offers a practical foundation for the responsible development of LLM-based mental health support. By outlining a layered architecture and aligning it with established evaluation standards, this work offers guidance for developing AI tools that are technically capable, safe, effective, and ethically sound. Future research should prioritize empirical validation of the framework through the phased, comparative approach introduced in this paper.

## Introduction

### Significance of Mental Health and Technology Integration

This paper presents a conceptual framework and an engineering viewpoint. It aims to guide the responsible and effective development of large language model (LLM)–based chatbots for mental health applications by synthesizing interdisciplinary research, proposing a structured approach to prompt engineering (PE), and outlining key design principles and ethical considerations essential for artificial intelligence (AI)–supported care. Our work draws upon the evolving understanding of LLM capabilities and the imperative need for frameworks that ensure safety, efficacy, and user trust in this sensitive domain [1,2]. Central to our framework is a commitment to a meaningful, long-term, interdisciplinary collaboration. This means involving mental health clinicians, clinical practitioners, ethicists, and other relevant domain experts from the earliest stages and throughout the entire development lifecycle. Such continuous engagement ensures that the development process remains firmly grounded in clinical evidence, ethical rigor, and contextual sensitivity, extending from initial conception to final deployment.

Mental health disorders remain a major global health concern, significantly contributing to disability and premature death [3]. According to the World Health Organization, more than 1 billion people experience some form of mental health or substance abuse disorder, costing trillions of dollars per year in economic expenses [4]. Access to timely interventions is also hindered by an absence of trained mental health professionals, widespread societal stigma, and limitations within health care systems [5,6]. In overcoming these barriers, digital health technologies hold great promise for enabling more accessible and equitable mental health care [7]. The progress in telemedicine, mobile health apps, and wearable sensors demonstrates the potential for continuous monitoring and treatment at home [8]. AI-powered mental health chatbots incorporating natural language processing and LLMs have demonstrated the capacity to provide scalable psychoeducation, emotional support, and skill training [9,10]. However, the effectiveness of chatbots depends on the quality of their prompt design, which controls their conversational style (tone and empathy), and adherence to clinical best practices [11].

### PE for Personalized and Effective Mental Health Chatbots

A prompt, when used with LLMs, refers to the user input that returns a particular output by the model [12]. Prompts can be static prompts—fixed, noneditable messages or instructions that remain the same regardless of user context or input. These are normally used for greeting, commands, or standardized queries. LLMs, or foundation models, are pretrained on massive text datasets to learn statistical patterns and structural characteristics of language [13]. Interpreted through the lens of prompts, users can interact with these models post training without altering their internal parameters. Despite the rapid advancements in generative models, such as OpenAI’s GPT-3.5 and GPT-4, most mental health chatbots still rely on static prompts (ie, fixed and predefined input formats) that produce predictable and impersonal dialogues [12,14].

To overcome these limitations, the practice of PE has emerged. PE is a structured, iterative process of designing and fine-tuning inputs to yield consistent, safe, and context-appropriate output from LLMs [15,16]. Beyond training alone, it incorporates principles from engineering disciplines. For mental well-being, this enables exact adaptation of domain-general LLMs to clinically sensitive processes so that AI-produced responses are not only coherent but also therapeutically meaningful and ethically sound [1,16].

Furthermore, effective PE supports the integration of evidence-based psychotherapies, such as cognitive behavioral therapy (CBT), acceptance and commitment therapy (ACT), and dialectical behavior therapy (DBT) into conversational AI frameworks [17-19]. This enables not only therapeutic accuracy but also safety-oriented functionality, like empathetic interaction, user retention, and crisis management. To mitigate potential risks, we advocate for end-to-end approaches that integrate user modeling, clinical objectives, clinician input, continuous feedback loops, and clearly defined ethical boundaries throughout the prompt lifecycle [20].

Recent research also shows that PE can enhance the efficacy of AI in high-stakes tasks through optimizing narrative coherence, cultural sensitivity, and linguistic adaptability [21-23]. For example, well-calibrated prompts can reduce cultural hallucinations in multilingual settings and refine emotional sensitivity in chatbots. Furthermore, real-time adaptation using user feedback enables adaptive refinement, further enhancing the reliability and trustworthiness of AI-aided mental health therapies [24].

### Contributions and Paper Organization

The central research problem addressed in this paper is the limitation of current AI-driven mental health chatbots, which often lack the personalization, clinical nuance, and safety protocols required for high-stakes applications. In response, this paper makes 3 main contributions. First, we propose the Mental Well-Being Through Dialogue – Safeguarded and Adaptive Framework for Ethics (MIND-SAFE), a layered conceptual framework that integrates evidence-based therapeutic models, adaptive technologies, and structured ethical safeguards. Second, we outline a comparative validation strategy that moves beyond a standard randomized controlled trial (RCT), assessing the framework’s added value while mapping its components to established evaluation standards such as the Framework for AI Tool Assessment in Mental Health (FAITA-MH) [25] and Readiness Evaluation for AI-Mental Health Deployment and Implementation (READI) [26] frameworks. Third, by presenting this framework, we provide practical guidance and principles for the responsible design of next-generation AI mental health chatbots.

The remainder of this paper is organized to build upon these contributions. The Methods section begins by establishing the conceptual foundations for the work and then details the proposed architecture of the MIND-SAFE framework. Subsequently, the Results section presents the framework itself as the primary output of this paper. Finally, the Discussion examines the framework’s implications for responsible AI development, its limitations, and directions for future research before the paper concludes.

## Methods

### Conceptual Foundations

#### Evolution of AI in Mental Health

Early applications of AI in mental health were primarily based on rule-based expert systems offering standardized advice with minimal flexibility in adjusting to individual users [27]. Early programs like ELIZA, developed in the 1960s, created the illusion of conversation through predefined scripts but did not involve real understanding or personalization [28]. Subsequent generations of machine learning (ML) and deep learning techniques extended capabilities by learning from enormous collections of user language, enabling simple sentiment analysis and symptom trend detection [29]. For example, ML models have been trained to detect depression from social media status updates and mental health crises from usage behaviors. This data-driven approach allowed for observations that transcended the fixed reasoning inherent in earlier expert systems, offering a more dynamic and potentially insightful analysis of mental states [30]. AI models have increasingly demonstrated capabilities to map human behavior and assist mental health practitioners with treatment and diagnosis [31].

The emergence of modern LLMs represents a paradigm shift in the application of AI to mental health. Building on earlier ML approaches, recent research shows that advanced LLMs (such as GPT-3.5 and GPT-4) can generate responses that are more empathetic and contextually relevant to users’ concerns than earlier chatbot paradigms [32]. These models leverage knowledge from massive training corpora, enabling them to understand nuanced language, recognize emotional cues, and even emulate elements of counseling styles [33]. A recent systematic review found that LLMs may demonstrate aspects of cognitive empathy, such as accurately recognizing users’ emotions and responding supportively across contexts [34]. Importantly, this ability is achieved through advanced pattern recognition rather than genuine emotional experience. In contrast, affective empathy involves the actual experience or sharing of emotions, which LLMs do not exhibit. Thus, current LLMs demonstrate cognitive (recognition) empathy, but not affective understanding [1,2]. In health care applications, studies have shown that AI-driven chatbots incorporating elements of cognitive empathy can enhance patient-physician interactions by improving perceived understanding and emotional support [35]. Furthermore, research suggests that AI chatbots can create the illusion of empathy, shaping user perceptions of conversational depth and emotional understanding [36]. Affective computing research has further reinforced the role of LLMs in enhancing empathetic interactions, demonstrating how these models can fine-tune responses based on emotional cues [37]. In some evaluations, an LLM-based chatbot’s responses were even preferred over human responses for empathy and support in online patient queries [38]. These findings underscore the potential of LLMs to enhance user engagement through empathy, a quality previously considered unique to human therapists [39]. Nevertheless, one should distinguish between cognitive and affective empathy. Current LLMs predominantly demonstrate the former. They can identify or “perceive” feelings but are not capable of experiencing them themselves, whereas affective empathy remains unique to humans [40].

Beyond text-based interactions, the field of AI for mental health is increasingly exploring multimodal models and affective computing approaches to enhance treatment. Affective computing is defined as the capability of computers to recognize, interpret, process, and simulate human emotions [41]. This involves the interpretation of multimodal signals, such as monitoring vocal tone, facial expressions (using facial emotion recognition), or physiological signals (heart rate and sleep patterns), to infer a user’s emotional state [42]. By tracking such feedback, AI can become more responsive to user well-being and adjust interventions accordingly [43]. For instance, a chatbot equipped with affective sensors could detect growing anxiety in a user’s voice and modify its responses to be less jarring or initiate grounding techniques [44]. This form of multimodal feedback holds the potential for real-time emotion context awareness, enabling interventions to be more tailored to the user and responsive to immediate needs [45]. This approach aligns with the vision that emotionally personalized interventions can improve the engagement and efficacy of online mental health treatment [46]. Emerging systems are beginning to adopt these concepts; for example, pilot chatbots use camera data for facial emotion recognition or prompt users to rate their mood via wearables, thereby integrating dialogue with objective mood data to better guide interactions [47]. Multimodal affective approaches, combining visual, audio, and text signals, are showing promising results in accurately identifying patient emotions, surpassing the outcomes of unimodal analysis [48].

Despite these advancements, significant limitations persist, and the translation of research innovations into practical, real-world applications often lags behind the pace of research [49,50]. Early iterations of mental health chatbots, such as Woebot and Wysa, predominantly relied on predetermined conversational scripts and decision trees [51]. While these systems were capable of assigning CBT homework and providing psychoeducation, they lacked the capacity for high-level adaptability to individual user contexts [15]. Consequently, users often received uniform, generic encouragement or guidance, irrespective of their unique backgrounds or the complexity of their inputs [52]. This rigidity likely contributed to inconsistencies in user responses and engagement [53]. These early chatbots, while offering preliminary support, were not designed to replace the in-depth, personalized care provided by human therapists and often struggled to interpret nuanced user responses [54]. They also lacked genuine human empathy, a crucial element in addressing complex emotional issues [55]. Furthermore, they were limited in the scope of interventions they could provide and were not equipped to handle severe mental health conditions requiring specialized care [56]. Ethical concerns also arose regarding unvetted feedback and potential misdiagnosis [57].

Recent developments in LLM-based chatbots offer the promise of more dynamic and contextually sensitive interactions; however, the integration of advanced models does not represent a complete solution [23]. A persistent challenge lies in effectively guiding LLMs to ensure that they provide assistance that is not only clinically appropriate and safe but also adheres to the highest standards of therapeutic practice [58]. Without proper guidance, an LLM might generate responses that, while linguistically fluent, are clinically suboptimal or potentially hazardous, for example, offering erroneous advice or failing to adequately address critical signs of suicidality [59]. Empirical evidence suggests that clarity and specificity in real-time interactions are crucial for fostering therapeutic rapport and enhancing user compliance during chatbot-facilitated interventions [60]. Yu and McGuinness [61] argue that the clinical efficacy of an LLM-based counselor is contingent upon the quality of its training; inferior prompts are prone to yielding imprecise or irrelevant outputs, whereas high-quality prompts tend to facilitate more effective and supportive dialogue. AI systems, while capable of identifying patterns, often struggle with the complexity and variability inherent in many mental illnesses [62]. The accuracy of AI in mental health also depends heavily on the quality and diversity of the data it is trained on, with incomplete or biased datasets potentially leading to diagnostic errors, particularly in diverse populations [63].

Furthermore, although state-of-the-art models are capable of emulating empathetic responses, they fundamentally lack genuine comprehension and accountability. They also exhibit notable issues with consistency and reliability within mental health contexts [49]. For example, LLMs can occasionally produce what might be termed “artificial empathy,” responses that, while seemingly warm, may eventually be perceived as superficial or inauthentic when encountered repeatedly or when the conversational context shifts slightly [15]. Some users have reported that, over time, AI-driven support can appear shallow or overly repetitive, suggesting that these systems have yet to fully embody the authentic spirit of a human therapeutic alliance [51]. General-purpose LLM chatbots, such as unmodified versions of ChatGPT, pose considerable risks when applied to mental health care. They may, at times, generate factually inaccurate or culturally insensitive responses or fail to provide an appropriate reaction during crisis situations [57]. Recent user studies have further highlighted concerns related to trust, privacy, and the establishment of clear boundaries when individuals seek support from AI systems [64]. These findings underscore that the benefits offered by contemporary LLMs, such as improved fluency, a broader knowledge base, and an empathetic tone, must be complemented by robust guiding frameworks to ensure safety and efficacy [61]. The absence of genuine human empathy and ethical principles in AI chatbots remains a significant limitation, potentially affecting the crucial therapeutic alliance [65].

The field is increasingly recognizing that techniques like PE are critical for harnessing the strengths of LLMs while mitigating their shortcomings in therapeutic settings [47]. In summary, the role of AI in mental health is evolving rapidly, transitioning from basic rule-based applications to more sophisticated LLMs and multimodal agents [60]. Although the potential for scalable, on-demand support is unprecedented, achieving safe, personalized, and clinically sound outcomes necessitates addressing the crucial challenge of effective control and guidance [66]. This context lays the groundwork for a more rigorous examination of PE as a mediating strategy between the inherent potential of AI and the complex demands of mental health treatment [67].

#### Foundations of PE

The practice of PE has emerged as a pivotal method for directing the actions of AI models, particularly within the landscape of LLMs. Broadly defined, PE involves the deliberate creation of input statements or directives provided to a model with the aim of eliciting the desired output [13]. Unlike extensive model training or fine-tuning, which necessitate substantial annotated datasets and computationally intensive weight updates, PE operates post training by carefully managing the contextual environment in which the model functions [68]. Through the meticulous crafting of questions, instructions, or contextual prompts, practitioners can optimize the likelihood of a pretrained model performing specific tasks or adopting particular response patterns [61]. In practice, PE has emerged as a state-of-the-art technique for adapting powerful general-purpose LLMs to specialized domains without requiring resource-intensive retraining processes [68]. Essentially, PE serves to “program” the model using natural language rather than traditional code. This burgeoning field focuses on designing and optimizing prompts to guide AI models, enabling them to understand intent, follow instructions, and generate desired outputs, acting as a roadmap for the AI to steer it toward a specific output [69].

In the context of mental health applications, PE presents both considerable promise and significant challenges. In contrast to simpler tasks, such as factual research or summarization, therapeutic conversations require a consistent maintenance of an appropriate tone, empathetic engagement, adherence to clinical standards, and strict ethical boundaries [70]. Prompts designed for mental health chatbots should therefore be designed to serve multiple therapeutic objectives concurrently: providing psychological education, offering emotional validation and empathy, and guiding users through evidence-based treatments, such as CBT thought reframing or guided breathing exercises, all while maintaining a personalized and supportive communication style [60].

For example, an effective prompt needs to inform the AI which therapeutic model to use (eg, CBT or mindfulness-based therapies), dictate the desired tone and readability of the communication, and enforce necessary constraints, such as avoiding trigger language or reporting unverified medical information [53,60,68]. The incorporation of robust foundation therapy models within prompts is a fundamental principle. Recent research has explored the transformation of techniques from CBT [71], ACT [72], and DBT [73] into formal prompt structures. Operationally, this might involve instructing the AI to induce cognitive distortions in a manner consistent with CBT principles or to guide users toward value-based actions according to ACT procedures. For instance, a novel prompting strategy inspired by DBT has demonstrated significant improvements in response accuracy on smaller LLMs by translating key DBT skills like Wise Mind, Observation, Description, and Effectiveness into prompt components [74].

#### Ethical and Regulatory Landscape

The ethical and regulatory considerations surrounding AI-driven mental health chatbots are of significant importance due to the sensitive nature of user data and the potential impact on individual well-being [75]. Legal frameworks such as the Health Insurance Portability and Accountability Act (HIPAA) in the United States and the General Data Protection Regulation (GDPR) in the European Union mandate stringent privacy and data protection protocols, including end-to-end encryption, secure data handling practices, and the provision of user rights regarding data control [76]. HIPAA primarily applies to covered entities like health care providers and business associates handling protected health information, consisting of details such as medical histories and mental health conditions [76,77]. GDPR, on the other hand, applies more broadly to the processing of personally identifiable information of EU residents, requiring organizations to safeguard data and provide documentation of protection protocols, including obtaining explicit consent and facilitating the “right to be forgotten” [76,77]. The increasing use of LLMs in health care, as discussed by Chow et al [2], further underscores the critical need for robust patient privacy and data security measures, particularly given the volume and sensitivity of data processed by LLM-enabled medical chatbots.

Privacy concerns are only one part of the equation; algorithmic bias and the inherent lack of explainability in some AI models present significant challenges [78]. Bias in training data can lead to disparities in care, potentially reinforcing societal stigmas [79]. This necessitates the development of “fair-aware AI” through proactive bias audits and mitigation strategies. The “black box” nature of certain AI algorithms can erode user trust and complicate accountability, underscoring the need for transparent usage policies and explainability features to foster user confidence and responsible governance [80]. For example, biases have been observed in AI models predicting Intensive Care Unit mortality and psychiatric readmission based on factors like gender and insurance type [81]. While general regulations like HIPAA and GDPR provide a foundational legal framework, specific deployment contexts, such as integration with existing health care systems or crisis services, will impose additional technical and compliance requirements. These may include specific data interoperability standards, security audit trails, and protocols for seamless escalation to human responders that align with the deploying organization’s workflows and risk management policies.

Maintaining safety and accountability is essential, particularly in situations involving potential crises. Ethical deployment requires robust safety controls, including clear escalation procedures for users reporting suicidal ideation, potentially involving human oversight [82]. The disruptive nature of advanced LLMs like ChatGPT, as highlighted by Chow et al [2], introduces new complexities, including concerns about the accuracy of information, potential for misuse, and the rapid pace of development outstripping current ethical and regulatory frameworks. This requires an urgent call for robust ethical frameworks and continuous vigilance to address the unique challenges posed by such powerful, general-purpose models when applied to sensitive domains like mental health. Regulatory efforts are increasingly classifying health-related AI as high-risk, demanding rigorous testing for bias, reliability, and safety [83]. Implementing ethics effectively necessitates continuous monitoring, user feedback mechanisms, and iterative refinement, adopting an “ethics by design” approach that involves ethicists and clinicians from the outset [84]. Such a collaborative stance aims to ensure that AI-driven mental well-being interventions are not only innovative but also ethically sound and deserving of consumer trust [85]. Principles of responsible AI emphasize human supervision, fairness, transparency, privacy, safety, security, professional responsibility, and accountability [86]. Ethics of care considerations for AI in mental health include respecting human dignity, refraining from abusing user trust or manipulating emotions, and acknowledging the cultural diversity of emotional expression [86].

While AI holds significant promise in revolutionizing mental health care, it is crucial to address the associated ethical challenges. Safeguarding patient confidentiality and trust requires careful management of privacy concerns related to the collection and analysis of sensitive personal data. Furthermore, the inherent biases present in AI algorithms can contribute to disparities in diagnosis and treatment, disproportionately affecting marginalized or underserved populations. To ensure AI is integrated safely and effectively into mental health counseling, ongoing research, transparency, and accountability must remain a key priority.

#### Gaps in Current Approaches

Current AI-powered mental health support systems, while offering convenience, exhibit significant shortcomings in their therapeutic impact due to several key gaps [87]. One primary limitation is the inability to provide nuanced personalization and responsiveness. Many chatbots rely on preformulated conversational flows and deterministic response patterns that fail to adequately consider unique user contexts, dynamic emotional states, or individual treatment experiences [88]. This superficial level of personalization, coupled with limited real-time user state monitoring and longitudinal adaptation, undermines the potential for long-term user engagement and the development of a strong therapeutic bond [89]. While AI holds the promise for hyperpersonalized treatment through the analysis of diverse data [90], current systems often struggle with the complexity and variability of mental illnesses, including the subtleties of human communication [91].

Furthermore, crisis management capabilities in current AI systems remain rudimentary, often limited to directing users to hotline phone numbers without incorporating comprehensive risk assessment or safety planning [92]. Although advancements are being made in using AI for crisis prediction, immediate support, and enhanced hotline systems [93], a comprehensive integration of these technologies into robust safety protocols is still needed. These technical limitations are compounded by a broader lack of robust clinical validation. Many AI tools in mental health have demonstrated limited evidence of effectiveness across diverse populations and in naturalistic settings, thereby hindering user trust and the integration of these technologies into established mental health care models [94,95]. While some AI-powered assessment tools are undergoing clinical validation [96], more extensive and diverse validation studies are necessary to ensure their reliability and efficacy. Addressing these critical gaps requires ongoing advancements in AI adaptability, personalized learning algorithms, and seamless integration with established clinical interventions to enhance the efficacy and reliability of chatbots in real-world applications [91,97].

The literature review in Table 1 highlights the significant evolution of AI in mental health, from early rule-based systems to sophisticated LLMs and multimodal approaches. While AI offers unprecedented potential for scalable and accessible mental health support, several critical challenges and gaps remain. The lack of genuine empathy, issues with safety and accountability, the presence of algorithmic bias, and limitations in personalization and crisis management necessitate careful consideration and further research. PE emerges as a crucial strategy for guiding LLMs to provide safe, ethical, and clinically relevant support. The ethical and regulatory landscape requires continuous attention to ensure user privacy, data security, and responsible AI implementation. Addressing the identified gaps through ongoing advancements in AI, rigorous clinical validation, and thoughtful integration with human expertise will be essential for realizing the full potential of AI in transforming mental health care.

**Table 1.** Evolution of artificial intelligence in mental health.

Era or technology	Key characteristics or capabilities	Examples	Limitations
1960s, rule-based systems or ELIZA [98]	Standardized advice and simulated conversation	ELIZA	Lack of personalization and no real understanding
1980s-2010s, ML^a^/DL^b^ [38]	Data-driven analysis, sentiment detection, and symptom trend detection	Depression detection from social media	Requires large datasets and potential for bias
2020s, large language models [15]	Empathetic responses, nuanced language understanding, and cognitive empathy	GPT-3.5 and GPT-4	Lack of genuine comprehension, safety concerns, and potential for superficiality
Present or future, multimodal AI^c^, and affective computing [41]	Emotion recognition from multiple inputs and personalized interventions	Chatbots using facial recognition and wearables	Technical complexity, ethical considerations, and need for robust validation

a ML: machine learning.

b DL: deep learning.

c AI: artificial intelligence.

#### Current Landscape of AI Chatbots in Mental Health and PE Limitations

The integration of AI in mental health intervention systems has the potential to contribute both benefits and risks. Table 2 provides an overview of the current landscape of AI chatbots applied to mental health interventions, and this is specifically in their therapeutic positions and the constraints placed on their quick engineering approaches. This assessment synthesizes the main characteristics of representative AI chatbots used in this field, as presented in Table 2. It demonstrates various AI chatbots used in mental health care, defining their treatment scope, cueing strategies, target populations of users, and the most critical limitations affecting their performance. This structured evaluation provides insight into the strengths and weaknesses of current AI-based mental well-being tools and identifies key areas for improvement.

The data provided in Table 2 show trends in the current use of AI-driven chatbots for mental health treatment. One of the trends is the universal use of CBT and mindfulness-based interventions on most of the leading platforms, including Woebot, Wysa, Replika, Tess, Youper, Sanvello, and MindShift. This initial focus suggests a foundation strategy of reconfiguring established therapeutic practices into practical AI formats to provide users with systematic instructions for the treatment of anxiety, stress, and similar issues. Although current chatbots borrow from therapeutic concepts, they are also lacking in how well they can respond to the specific needs of individual users and tailor interactions.

**Table 2.** Representative artificial intelligence chatbots used in mental health interventions.

Chatbot	Therapeutic approach	Prompt strategies	Target audience	Limitations	Reference
Woebot	CBT^e^-focused, anxiety and stress management	Prescripted conversation flows	Adults	Limited adaptive personalization; minimal role assignment customization	Manole et al [50] and Fitzpatrick et al [99]
Wysa	CBT-focused, mindfulness, anxiety, and stress management	Empathetic but generic prompts	Teens, adults	Restricted context integration; lacks dynamic real-time updates	Inkster et al [100] and Chaudhry and Debi [101]
Replika	CBT-focused, mindfulness, general supportive talk	Passive, user-led queries	General	Inconsistent therapy alignment; uncertain crisis management features	AI Foysal [58] and Moylan and Doherty [102]
Tess	CBT and motivational interviewing	Thematically structured modules	General	Basic prompt cues; lacks deeper user-specific context analysis	Fulmer et al [103] and Stephens et al [104]
Talk space Chatbot	Guided self-help and therapy access facilitation	Guided conversation flows with service integration prompts	General	Limited direct therapeutic intervention; primarily a gateway to paid services; not a replacement for therapy	Larson [105] and Anser et al [106]
Rasa^b^	Modular framework (open source)	Customizable NLU^c^ and dialogue flows; partial script expansions	Developers	Requires specialized design; does not inherently provide integrated therapeutic cues	Hanji et al [107] and Vineeth et al [108]
Youper	AI^d^-driven CBT and mindfulness	Adaptive daily check-ins	General	May lack advanced role assignment; personalization limited to user mood surveys	Mehta et al [109] and Major study from Stanford University and Youper find artificial intelligence therapy effective at reducing anxiety and depression [110]
Sanvello	CBT, mindfulness, relaxation	Prebuilt guided journeys and journaling prompts	General	Primarily static prompts; expansions often generic, lacking advanced user context	Balaskas et al [111] and Bautista et al [112]
MindShift	CBT for anxiety	Topic-focused modules, psychoeducation	General	Narrow domain focus (anxiety); conversation logic not strongly customized	Garrido et al [113] and Sharma et al [114]
Ada AI	Symptom checker+chat support	Basic triage-style prompts; limited mental health focus	General	Lacks robust therapy alignment; prompt structure is primarily biomedical	Morse et al [115] and Jungmann et al [116]

a CBT: cognitive behavioral therapy.

b Rasa is a framework rather than a standalone chatbot; it allows building custom bots with its natural language understanding and dialogue management, so the prompt strategy depends on the implementation.

c NLU: natural language understanding.

d AI: artificial intelligence.

A typical problem highlighted in the analysis is limited adaptive personalization and restricted context integration across various chatbots. Phrases like “Limited adaptive personalization” and “Lacking deeper user-specific context analysis” identify the difficulty of developing AI systems that can completely understand and respond to the intricacies of a person’s mental health process. This is, in part, due to the current PE methods used, which are centered around predefined dialogue flows, generic prompts, or modular designs. As much as these offer control and guidance to some extent, they may fail to adequately respond to new user inputs or venture into special issues and, therefore, may make the conversation unnatural and limit the chatbot’s capacity to offer highly tailored support. Even modular designs like Rasa, while being customizable, require expert knowledge to integrate therapeutic goals and user specificity into the prompt strategy efficiently.

To overcome these limitations, a radical overhaul of PE is required. Future generations of techniques must aim to build end-to-end architectures that can understand user intent and emotion more deeply, potentially by leveraging multimodal data and building dynamic user models that learn and adapt over time. Integrating established therapeutic principles directly into these frameworks, with cultural sensitivities, and exploring human-AI collaboration in therapy are also crucial. Finally, rigorous evaluation frameworks will be required to demonstrate the efficacy, safety, and ethical implications of these new AI mental health technologies, resulting in more advanced and effective therapies.

As Table 2 suggests, despite advancements in AI-driven mental health support, current systems demonstrate limitations in adaptivity and personalization, hindering their capacity to deliver truly nuanced and effective therapeutic interventions. While frameworks like Rasa enable technical customization, achieving systematic integration of therapeutic goals and user-specific nuances remains a significant challenge. Future research should prioritize holistic PE frameworks that synergize therapeutic best practices with AI capabilities, focusing on real-time user adaptation through multimodal signal processing, dynamic personalization via evolving user models, and cultural adaptation to ensure global applicability. Furthermore, exploring human-AI collaborative models and establishing rigorous evaluation frameworks are crucial to bridge existing gaps, quantify improvements, and ultimately advance AI interventions that meaningfully complement mental health care with enhanced personalization, safety, and efficacy.

### Proposed Framework

#### Overview

**Figure 1.**
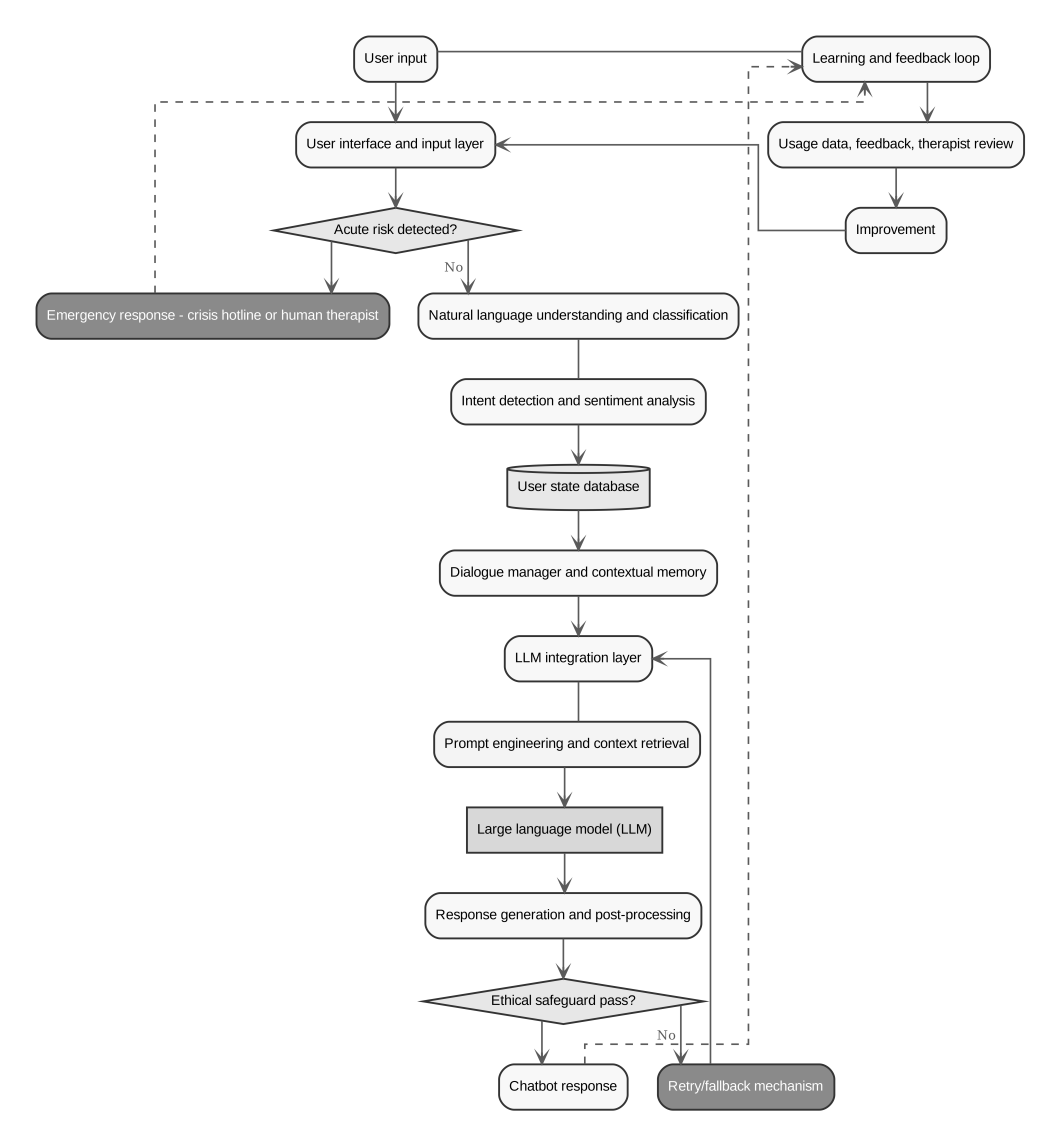
The architecture of the MIND-SAFE framework.

The MIND-SAFE framework is introduced as a structured approach to integrating LLMs into an AI-driven mental health chatbot system. As illustrated in Figure 1, the proposed architecture emphasizes user adaptation, ethical safeguarding, and personalized intervention, providing a foundation for responsible deployment in mental health contexts. Figure 1 illustrates the architecture, demonstrating how the user input flows through the linear layers of processing, protection, and response generation. Unlike conventional single-stage chatbots, our architecture introduces layered safety mechanisms (Figure 1) to detect acute risks and mitigate biases. This multitier design not only prioritizes user well-being but also incorporates a dedicated user state database (USD) for personalized intervention while protecting data privacy.

General chatbot architecture in the proposed framework is adapted from a design in a study by Chow et al [117]. The system combines user interaction, LLM-based dialogue generation, and multitiered safety controls to deliver personalized mental health support. The flowchart illustrates the architecture’s main components and data flow. Beginning with the user’s input (which undergoes risk checks), the process moves through intent analysis and dialogue management, then to the LLM with layered prompts (including ethical instructions and retrieved knowledge). The LLM’s output is filtered for safety before being presented as the chatbot’s reply to the user. A feedback loop with expert oversight and user data analytics continuously updates the system for improvements (Figure 1). The following sections detail the system’s unique components and their roles in maintaining ethical and clinically guided interactions. At a high level, the system is composed of the following key components:

#### User Interface and Input Layer

The initial stage, the “user interface” and “input layer” (eg, a chat window or mobile app), focuses on capturing the user’s expressions or questions (eg, “I feel hopeless and anxious”) through a conversational interface. This layer does minimal preprocessing (language identification and tokenization) and flags a message as an acute risk immediately if any sign of acute danger (eg, self-harm cues) is detected via keyword spotting or a basic classifier. To operationalize immediate crisis detection, this layer combines keyword spotting (eg, “I want to end my life”) with a lightweight risk classifier inspired by standard clinical screening tools. Each incoming user message is assigned a risk score based on the severity of identified linguistic cues. If a threshold is exceeded, the system promptly routes the user to emergency response (bypassing the LLM), aligning with guidance from professional bodies (eg, American Psychological Association [APA]) on crisis handling.

This threshold-driven approach aims to minimize false negatives and ensure that no potentially harmful generative output is offered during emergency scenarios. When a crisis has been detected (eg, overt suicidal thinking), the system initiates an emergency response (eg, providing crisis hotline numbers or pinging a human therapist) before any engagement with an LLM. This proactive safety measure aligns with the concerns raised by the APA regarding the potential dangers of unsupervised AI chatbots in mental health support [118]. The APA emphasizes the necessity of safeguards to protect the public from harm, particularly in vulnerable situations. The framework’s commitment to prioritizing user safety in high-risk scenarios before engaging the LLM demonstrates a responsible approach to the clinical use of AI.

#### Natural Language Understanding and Classification

Once an input is considered safe for automated processing, the next module identifies the user intent and emotional sentiment. While modern LLMs are capable of performing intent detection and sentiment analysis in context on their own, the framework proposes augmenting this with smaller classifiers for more specialized tasks, such as distinguishing between depressive mood, anxiety, or general advice. For example, negative self-talk could trigger a cognitive restructuring strategy, and a request for motivation might initiate a behavioral activation strategy. This steering mechanism, which uses auxiliary models or prompt-based classification, aims to improve both the relevance and safety of the interaction [119]. The design and validation of these specialized classifiers, as well as the interpretation of their outputs for the USD, will be conducted in close collaboration with clinical psychologists to ensure alignment with established diagnostic criteria and therapeutic relevance. Furthermore, the classifier is designed to update a user profile (eg, “user tends to exhibit social anxiety”), maintained in what we term the USD, to facilitate personalization and maintain conversational context.

The USD is a secure, structured repository specifically designed within our framework to store anonymized key information derived from user interactions. This includes aggregated sentiment trends, identified emotional triggers (eg, recurring themes associated with anxiety), preferred coping strategies previously discussed, and progress on therapeutic exercises. It is not a verbatim log of conversations but rather a distilled representation of the user’s journey and state, enabling the chatbot to maintain continuity across sessions, personalize responses (eg, recalling a previously effective grounding technique), and adapt its approach based on the user’s evolving needs. The USD operates under strict data minimization principles, retaining only essential, deidentified attributes necessary for therapeutic adaptation, thereby adhering to privacy standards such as HIPAA and GDPR, as discussed by Chow et al [2] in the context of LLM-enabled health care.

This data-minimization approach of the USD upholds privacy standards (eg, HIPAA and GDPR) and mitigates reidentification risks. Access control mechanisms ensure that only authorized clinicians or system modules can read or modify user states, while encryption at rest and in transit safeguards sensitive mental health data. By centralizing user states rather than entire dialogue logs, the USD fosters consistent personalization (eg, recurring self-talk patterns) without compromising confidentiality. This layered approach, using the strengths of both general LLMs and specialized classifiers, suggests an efficient allocation of resources and an enhanced ability to address specific user needs.

#### Dialogue Management and Contextual Memory

The dialogue management and contextual memory component is central to conducting coherent and therapeutically aligned conversations. Working in close concert with the clinical experts, this module synthesizes the current dialogue history, relevant information from the USD, and any necessary external information to construct a comprehensive context. This preconstructed context is then passed to the LLM integration layer. A carefully constructed system prompt, which is co-designed with therapists, defines the LLM’s role (eg, a supportive mental health assistant using cognitive-behavioral techniques) and establishes strict instructions regarding confidentiality, tone, and boundaries, such as avoiding medical diagnoses or inappropriate advice. This system-level prompt, reflecting clinical expert input, acts as an inherent ethical governor, ensuring the LLM’s outputs align with established therapy methods and ethical codes, including the avoidance of harmful suggestions. This co-design process includes translating evidence-based therapeutic techniques (eg, Socratic questioning, behavioral activation, and empathic reflection) into precise prompt structures. In addition, the LLM receives the current dialogue context, including recent user and chatbot messages, along with any relevant personal context or knowledge.

A key function of this layer is to determine when to leverage external knowledge through a retrieval-augmented generation (RAG) mechanism. For example, based on the conversational context, it can decide to query a knowledge base of verified psychological strategies or the user’s own journal entries. This retrieved information is then passed to the LLM integration layer to be incorporated directly into the prompt. This process, co-designed with clinical experts, is important for grounding the LLM’s response in evidence-based, contextually relevant content, thereby reducing the risk of factual inaccuracies or “hallucinations” and enhancing the therapeutic value of the dialog [120,121].

Existing mental health chatbots often rely on a single LLM pipeline with limited oversight. In contrast, our proposed layered architecture integrates PE, specialized classifiers, and a multistep ethical safeguard filter, creating a modular framework that can dynamically adapt or escalate when high-risk content arises. Unlike vanilla generative chatbots, this design ensures evidence-based content retrieval (eg, validated coping strategies), thus enhancing both safety and factual accuracy [121]. The iterative review and refinement of these dialog strategies and prompt structures by therapists, as part of the Learning and Feedback Loop section, is crucial for maintaining therapeutic fidelity and safety, aligning with recommendations by Stade et al [1] for responsibly tailoring LLMs.

#### LLM Integration Layer

The LLM integration layer is where the core therapeutic dialogue is generated. The framework uses PE to guide the LLM’s behavior within ethical and clinical bounds. A carefully crafted system prompt encodes the role (eg, “You are a supportive mental health assistant employing cognitive-behavioral techniques”) and strict instructions about confidentiality, tone, and boundaries (for instance, avoiding any medical diagnoses or inappropriate advice). This system-level prompt acts as an implicit ethical governor, ensuring the LLM’s outputs align with established therapy methods and ethical codes (such as never encouraging harmful behavior). In addition, the LLM is provided with the dialog context (recent user and chatbot messages) and any retrieved personal context or knowledge (described below) as part of the prompt. Figure 1 depicts this flow, where the user’s query, contextual data, and system instructions are combined and fed into the LLM.

#### Response Generation and Postprocessing

The raw output from the LLM then undergoes processing by the response generation and postprocessing module, which includes an ethical safeguard filter before being presented to the user. This filter verifies the LLM’s output for any violations of predefined rules or the presence of obscene language. While many recent LLM implementations include their own toxicity filters, the framework proposes an additional layer for filtering specifically tailored for mental health applications. This filter checks for compassionate language, the absence of derogatory or overly commanding speech, and the exclusion of banned content, such as information on lethal means or others’ personal identifying information. If the LLM’s output is flagged, the system may attempt to regenerate the response with an updated prompt or revert to a safe, predefined template. This layered safety approach demonstrates a strong commitment to ensuring that users receive clinically safe and high-quality responses, even if the generative model initially produces an unsuitable output. Furthermore, this filter is integrated with an explanation module that logs any changes made to the response, providing transparency for developers and accountability for clinicians reviewing the transcripts.

In addition, if the user’s ongoing profile in the USD indicates repeated exposure to triggering content, the system can escalate to an alternative intervention (eg, human therapist review or a predefined safe response). This layered postprocessing, a key feature of our proposed framework, bolsters consistency in mental health guidance and reduces the risk of repetitive, harmful outputs.

#### Learning and Feedback Loop

The final key component of the system is the learning and feedback loop, which facilitates continuous improvement over time. The system logs all user interactions in a secure and anonymized manner. Trained therapists periodically review these data to assess the chatbot’s effectiveness in adhering to therapeutic techniques and ethical norms, identifying areas for potential enhancement, such as improving the chatbot’s empathy. The “therapist review” component is important. Licensed clinicians will regularly audit anonymized interaction logs to assess adherence to therapeutic principles, identify areas for prompt refinement, and flag any instances of suboptimal or potentially harmful AI behavior. Their expertise is vital for interpreting nuanced interactions that automated metrics might miss. User feedback and ratings also contribute to a learning module that adjusts the chatbot’s operational parameters, potentially modifying question construction based on user engagement metrics like drop-off rates. This human oversight and user-driven feedback mechanism is important for ensuring the chatbot remains aligned with therapeutic best practices and user needs, addressing concerns about the potential lack of human empathy in AI interactions [122] and the necessity of continuous monitoring and improvement in AI systems [123].

As part of the learning and feedback loop, we propose a structured empirical evaluation strategy.

First, pilot tests in which small-scale user studies with diverse demographic groups are conducted to assess immediate safety, bias detection, and user experience.

Second, clinical trials in which collaborative studies with licensed therapists will be essential for evaluating therapeutic outcomes. For a rigorous evaluation of efficacy, particularly for specific mental health concerns targeted by the framework, these trials should ideally be designed as RCTs. This involves comparing the chatbot intervention against appropriate control groups and using a comprehensive suite of outcome measures, including but not limited to self-report, clinician-rated assessments, and behavioral data. Such rigor is fundamental to validating the framework’s effectiveness, especially if interventions derived from it are to be considered for users with diagnosed mental health conditions.

Third, longitudinal monitoring in which ongoing analyses of anonymized transcripts are used to identify any emergent biases or failures in acute-risk detection. Therapist oversight ensures that flagged dialogs receive expert attention, and the LLM’s prompts are recalibrated as necessary.

Fourth, user satisfaction and drop-off metrics that are collected via surveys and chatbot usage analytics feed into the iterative updates of prompt templates and dialogue strategies.

These steps aim to ensure that the chatbot not only meets immediate safety standards but also demonstrates long-term effectiveness in diverse real-world contexts [122,123].

The proposed framework has its limitations. This framework is designed to guide PE in AI mental health applications through the integration of therapeutic principles, user-centered design, and ethical considerations. However, it is not a catch-all solution and does not replace the need for clinical validation before real-world deployment. Such validation must assess the model’s interpretability, the quality and representativeness of its training data, and its fine-tuning for mental health contexts. While the framework can help identify effective PE methods, it does not guarantee their feasibility or success in clinical settings. Furthermore, given the rapid evolution of LLMs and shifting cultural norms, these limitations may change, requiring ongoing review and refinement of the framework.

### Ethical Architecture and Safeguards

While the previous section detailed the technical architecture of the framework, this section outlines the design principles and operational safeguards embedded within it to address core ethical responsibilities. These components are structured to systematically manage trust, privacy, fairness, and cultural inclusivity.

#### Privacy, Data Protection, and Regulatory Compliance

In mental health contexts, users may share highly sensitive information that requires enhanced security measures [77]. Our framework, therefore, adopts a privacy-by-design approach that emphasizes data minimization and strict access controls in the USD. This database only stores the essential states necessary to support personalization (eg, mood trends and recurring topics) rather than detailed transcripts or personally identifiable information. In alignment with HIPAA and GDPR regulations, the USD applies encryption for data at rest and in transit [77]. Session data is automatically purged or anonymized after a predefined period, ensuring that historical records do not accumulate beyond what is necessary for improving user experience [124].

The acute risk detection module flags user inputs suggestive of suicidal or violent ideation. When triggered, the system generates a minimal “risk event” log without storing the entire transcript. This design complies with telehealth regulations that discourage retaining sensitive health data unnecessarily [77,124]. In addition, postprocessing within the ethical safeguard filter ensures that the chatbot does not inadvertently echo confidential user details in its outputs.

#### Bias and Fairness

Bias in AI-driven mental health applications can lead to inequitable quality of support across demographic groups [125]. To address this risk, our framework incorporates a multitiered bias mitigation strategy. This approach begins with the foundational fine-tuning of the baseline LLM using demographically representative data, which includes targeted evaluations to detect potential bias in mental health–related responses [66]. Second, at the point of interaction within the LLM integration layer, prompt-level interventions are used; system instructions are explicitly designed to emphasize respectful, culturally neutral language, building on previous research showing that careful prompt design can significantly decrease discriminatory or stigmatizing responses [15,126]. Finally, the learning and feedback loop provides a mechanism for ongoing oversight. Human therapists will regularly review anonymized transcripts, specifically focusing on potential disparities in support across cultural or linguistic backgrounds. If persistent biases are identified, the dialogue manager can be adjusted, or underlying models can be retrained to better align with fairness objectives [127]. By combining these automated checks and dedicated therapist oversight, the system is designed to iteratively refine its interactions and promote equitable access to mental health support for all users.

#### Cultural Adaptation and Global Scalability

Developing culturally appropriate AI for global mental health requires more than just technical infrastructure; cultural competence is a critical element for successful implementation [23]. Cultural differences in expressing psychological distress, coping mechanisms, and attitudes toward mental illness are vast; therefore, interventions based solely on direct translation are likely to be culturally inappropriate or irrelevant [128]. For example, therapeutic metaphors based on environmental contexts unavailable in a user’s geographical setting may fail to resonate. Our framework design necessitates collaboration with local mental health practitioners to ensure the cultural relevance of intervention strategies by using appropriate examples and contextual schemas [129]. Piloting and obtaining user feedback in each cultural context is integral to the learning and feedback loop, allowing for the iterative refinement of both algorithms and intervention methods [130]. This methodological approach enhances the cross-cultural validity of therapeutic interactions, thereby augmenting user engagement and intervention usage [1]. The incorporation of culturally responsive elements into the system design provides support that aligns with the diverse experiences of various populations [131].

### A Strategy for Framework Validation

#### Overview

The validation of our proposed PE framework requires a multifaceted strategy that assesses its core components and their integrated performance. A full empirical validation is beyond the scope of this conceptual paper; however, this section outlines a phased plan that draws on best practices for evaluating AI in health care [1,26]. The primary objective is to demonstrate the framework’s ability to enhance the safety, clinical relevance, user engagement, and ethical integrity of LLM-based mental health chatbots compared with simpler implementations.

#### A Comparative Approach to Validating the Framework’s Value

To empirically assess the added value of the framework, a comparative evaluation is required. While a full RCT remains the gold standard for testing a specific, mature chatbot intervention, evaluating the framework itself necessitates a different approach. We propose a study comparing a chatbot built on the full, layered architecture against a minimally safe LLM baseline. This baseline would consist of a general-purpose LLM equipped only with the core safety mechanisms mandated by ethical practice: the acute risk detection module and the ethical safeguard filter. This ensures that no participant is exposed to an unsafe intervention, allowing the study to ethically isolate the therapeutic and engagement benefits of the framework’s more advanced components.

Such a design presents ethical challenges, as one cannot withhold necessary safety components. However, by ensuring a robust safety baseline in both arms, the comparison can ethically proceed to measure the incremental value of the framework’s other components, such as the USD, the specialized classifiers for intent detection, and the RAG mechanism. The key outcome measures would not be limited to clinical symptom reduction but would focus on intermediate metrics critical to the therapeutic process, such as the quality of the therapeutic alliance, perceived empathy, user trust, and the clinical relevance of the chatbot’s responses. This approach would enable us to isolate and quantify the specific benefits derived from the structured layered design, providing a clear evidence base for its utility compared with more basic implementations. A traditional RCT would then serve as the appropriate final step in a long-term research program, used to test a fully developed chatbot that has been refined through these earlier comparative studies.

#### Alignment With Established Evaluation Frameworks

Rather than introducing a new evaluation framework, which risks conceptual fragmentation, we demonstrate how the proposed framework (Figure 1) can be rigorously assessed using criteria from established, comprehensive evaluation frameworks in AI and mental health. Specifically, we align our validation strategy with the constructs outlined in the FAITA-MH [25] and the READI framework [26]. This alignment ensures that our approach contributes to a shared language and set of standards for responsible development.

The FAITA-MH framework provides a structured scale for evaluating AI-powered mental health tools across 6 dimensions: credibility, user experience, user agency, equity and inclusivity, transparency, and crisis management. Similarly, the READI framework outlines 6 key criteria for assessing the readiness of AI-mental health applications for clinical deployment: safety, privacy and confidentiality, equity, effectiveness, engagement, and implementation. Our framework components are designed to directly address these domains. Table 3 presents a mapping of each framework component to these standards, demonstrating how the design supports established evaluative criteria.

**Table 3.** Mapping of the proposed architectural components to constructs from the FAITA-MH^a^ and READI^b^ evaluation frameworks.

Our framework component (Figure 1)	Key function	Relevant FAITA-MH constructs	Relevant READI constructs
Acute risk detection and emergency response	Immediate crisis identification and escalation.	Crisis management and credibility	Safety
Ethical safeguard filter	Postgeneration check for safety, bias, and privacy violations.	Crisis management, and equity and inclusivity	Safety and privacy or confidentiality
User state database	Securely stores user state for personalization, and ensuring data minimization.	User agency (data protection and privacy) and user experience (personalized adaptability)	Privacy or confidentiality and effectiveness
Dialogue manager and LLM^c^ integration layer (with RAG^d^)	Generates clinically-grounded, empathetic dialogue using therapeutic models and verified knowledge.	Credibility (evidence-based content) and user experience (quality of interactions)	Effectiveness and Engagement
Natural language understanding and classification	Identifies user intent and sentiment to guide responses.	User experience (personalized adaptability)	Effectiveness
Learning and feedback loop (with therapist review)	Iterative improvement based on expert and user feedback.	Credibility (retention) and user experience (mechanisms for feedback)	Implementation and effectiveness
Entire framework	Holistic design integrating all components.	Transparency, equity, and inclusivity	Equity and implementation

a FAITA-MH: Framework for AI Tool Assessment in Mental Health.

b READI: Readiness Evaluation for AI-Mental Health Deployment and Implementation.

c LLM: large language model.

d RAG: retrieval-augmented generation.

#### Summary of Evaluation Methods

A systematic outline of the evaluation criteria is necessary for a comprehensive review of the performance and safety aspects of our proposed framework. Table 4 presents key dimensions and recommended metrics for evaluating chatbots built upon this framework. These dimensions and metrics are selected to align with and provide concrete measures for the core constructs of the FAITA-MH and READI evaluation frameworks, as detailed in Table 3. This includes assessing clinical effectiveness, user engagement, therapy quality, and ethical compliance. Building on this mapping, Table 4 specifies concrete evaluation dimensions, metrics, and tools aligned with FAITA-MH and READI.

As described above, the minimally safe baseline will undergo an identical assessment to evaluate outcome differences across equity, trust, and alliance measures. Analytically, outcomes will be assessed using preregistered analyses (eg, mixed-effects models for repeated measures and equivalence or noninferiority margins where appropriate).

**Table 4.** Core dimensions for evaluating artificial intelligence–driven mental health chatbots and recommended methods.

Dimension	Key metrics or methods	Primary objective	Tools or methods	References
Clinical efficacy	PHQ-9^a^, GAD-7^b^ symptom changes; RCT^c^ outcome comparisons	To assess reduction in mental health symptoms	Standardized questionnaires and pilot RCTs	Anisha et al [132]
User engagement	Session length, dropout rates, and exercise completion	To determine usage continuity and adherence	Chatbot log analytics and retention surveys	Liu et al [133]
Prompt quality	Likert-scale user feedback on empathy and clarity	To evaluate perceived helpfulness of generated responses	Postsession rating forms and chat reviews	Martinengo et al [134]
Bias and fairness	Comparison of responses across demographic groups	To detect and mitigate algorithmic bias	Stratified output analysis and bias audits	Torous et al [95]
Ethical compliance	Crisis protocol activation and privacy law conformance	To ensure user safety and regulatory adherence	IRB^d^ reviews and scenario-based crisis testing	Mennella et al [82]
Therapy quality	Therapist-coded transcripts and missed-opportunity logs	To validate adherence to therapeutic best practices	Structured auditing by mental health professionals	Vossen et al [135]
Cultural adaptation	Localization success and user satisfaction in multiple locales	To confirm the chatbot’s global relevance and inclusivity	International pilot studies and local feedback	Ulrich et al [136]

a PHQ-9: Patient Health Questionnaire-9.

b GAD-7: Generalized Anxiety Disorder 7-item.

c RCT: randomized controlled trial.

d IRB: Institutional Review Borad.

### Ethical Considerations

This paper presents a conceptual framework and synthesizes existing literature; it does not involve the collection or analysis of data from human participants, animals, or tissues. As such, and in accordance with GVSU's guidance on research activities not requiring Institutional Review Board (IRB) approval, the research was exempt from IRB or research ethics board approval [137]. All future research aimed at implementing and validating this framework, including the comparative evaluation strategy outlined in the "A Strategy for Framework Validation" section, will be subject to full ethics review and will require informed consent from all participants prior to their involvement.

## Results

The methodological analysis resulted in the development of the MIND-SAFE framework, a layered conceptual architecture for PE (Figure 1), designed to guide the creation of safe and effective LLM-based mental health chatbots. The principal result is the framework’s architecture, which systematically integrates evidence-based therapeutic models, a personalization layer with a USD and RAG mechanism, and a multitiered system of ethical and safety controls.

A further result of this work is the explicit mapping of the framework’s architectural components to establish evaluation standards, namely the FAITA-MH and READI frameworks (Table 3), which ensures alignment with best practices for responsible AI development. Finally, this work produced a systematic validation strategy, including core evaluative dimensions and metrics (Table 4), designed to empirically assess the framework’s value against a minimally safe baseline.

## Discussion

### Principal Findings

This integrated approach represents a methodological advance over existing systems, which often rely on scripted conversational flows or lack the governance needed for high-stakes clinical contexts [87,88]. As our review of the current landscape shows (Table 2), many chatbots provide limited personalization and inadequate context integration, restricting their ability to deliver nuanced support. By operationalizing principles of responsible AI development [1,26], our framework offers a structured pathway for creating more clinically relevant and trustworthy AI-driven therapeutic tools.

### Implications for Responsible AI Development

The proposed framework serves as a practical roadmap for developers, researchers, and clinicians seeking to move beyond general-purpose LLM deployments toward specialized, safer, and more clinically meaningful interventions. Its layered design addresses several core challenges in responsible AI.

First, the emphasis on a multitiered safety system, including both preprocessing risk detection and postprocessing ethical filters, establishes a defense-in-depth strategy against harmful or inappropriate outputs. This marks a critical departure from relying solely on internal safeguards of foundation models, which may not be sufficiently tailored to the sensitivities of mental health contexts. Second, the integration of the USD and a RAG mechanism provides a structured approach to personalization and factual grounding. This mitigates 2 major risks associated with LLMs in health care applications—the production of generic or clinically irrelevant responses, and the risk of factually incorrect outputs (“hallucinations”) [131].

Finally, the inclusion of a learning and feedback loop, which requires continuous therapist oversight, ensures that the system remains clinically grounded and can be iteratively refined. This human-in-the-loop model is not an auxiliary feature but a foundational principle, reflecting the ethical requirement that AI must augment, not replace, human clinical judgment and expertise. By operationalizing these principles, the framework provides a structured methodology for building AI systems that are not only technologically advanced but also aligned with the core tenets of safety, fairness, transparency, and accountability.

### Broader Ethical and Clinical Implications

The deployment of LLM-based mental health chatbots requires a proactive and rigorous approach to ethical governance. As noted by Chow et al [2], the rapid evolution of models like ChatGPT presents both opportunities and significant ethical challenges, including data accuracy, cybersecurity, patient safety, and the transparency of decision-making processes. The MIND-SAFE framework addresses these issues by embedding safeguards at multiple levels, from the user interface and input layer to the LLM integration layer and USD. This design prioritizes user trust, privacy, bias mitigation, and cultural inclusivity in each phase.

Establishing transparency is important in maintaining user trust. As illustrated in Figure 1, all interactions begin at the user interface and input layer, where the system explicitly discloses that the user is conversing with an AI chatbot. This disclosure clarifies the chatbot’s capabilities and limitations, reducing the risk of misunderstanding in situations that require clinical intervention. Furthermore, when the acute risk detection module detects or identifies suicidal ideation or crisis-level distress, the system bypasses the LLM and routes the user to emergency response or services. A targeted disclaimer reinforces that a licensed mental health professional or hotline is recommended in acute situations.

While such escalation protocols are a necessary safety net, they are insufficient compared with the ethical and legal responsibilities traditionally assumed by licensed professionals. A notable example is the “duty to protect,” a legal and ethical obligation established in Tarasoff v. Regents of the University of California (1976) [138]. This doctrine requires clinicians to take reasonable steps to prevent identifiable foreseeable harm, including breaching confidentiality to warn authorities or threatened victims. Because the scope and interpretation of this duty vary significantly across jurisdictions [138,139], even offering a hotline may not suffice in situations that legally or ethically require more active intervention.

Current AI systems cannot perform the nuanced legal reasoning or risk assessments required for such obligations [138]. This gap highlights the limitations of automation in contexts where professional accountability is essential [140]. One potential avenue for future development is the “warm handoff,” in which an AI system facilitates a direct, immediate transfer to a human crisis counselor rather than merely providing referral information [141]. Implementing this at scale presents significant technical, logistical, and workforce challenges and remains a critical area for research [100]. Accordingly, while our framework provides a robust technical safety net, we underscore that it is intended to augment rather than replace clinical oversight, particularly in high-risk scenarios where the duty to protect may be invoked [138,142].

### Limitations and Future Work

This paper presents the MIND-SAFE framework, a conceptual model intended to support the responsible, safe, and clinically meaningful development of AI-supported care platforms. While the framework is informed by interdisciplinary literature and sound design principles [1,2], its key limitation is the absence of empirical validation of the integrated system. The actual effectiveness and resilience of the proposed layered framework in real-world contexts remain to be determined through systematic testing, as outlined in the validation plan. This reflects the current stage of early development, where conceptual models remain high-level designs pending implementation and clinical validation. Our intention is to provide a structured, evidence-based, and ethically grounded framework to guide these next phases of research and evaluation.

Beyond empirical validation, the framework is also constrained by the current limitations of LLM technology. A central difficulty lies in conveying complex therapeutic nuance and achieving genuine affective empathy, rather than sophisticated pattern recognition or surface-level emulation of empathetic language [2]. Although the framework incorporates evidence-based therapeutic theory and engages with affective computing, replicating authentic empathy remains an unresolved challenge [1]. In addition, LLMs are prone to generating plausible but inaccurate information (“hallucinations”) and may reproduce or amplify biases present in their training data, even with mitigation strategies in place [26,134]. The effectiveness of the framework, therefore, depends heavily on the quality and diversity of both the auxiliary classifiers and the LLM training data; bias in either data or prompts could inadvertently lead to inequitable or suboptimal care. Continuous monitoring and iterative refinement are thus essential to safeguard fairness. The evolving nature of LLMs further implies that prompt design strategies and technical parameters will require ongoing adaptation as new models and capabilities emerge. Finally, while the framework’s use of a USD for personalization is based on privacy-by-design principles, its responsible implementation is critical to prevent data breaches and ensure compliance with evolving regulations such as HIPAA and GDPR [2]. The shifting regulatory environment for AI in health care demands continual oversight to maintain compliance and public trust [2,13,26].

Looking ahead, the vision of a clinically nuanced, adaptive, and ethically robust LLM-based mental health chatbot remains an ambitious, evolving research agenda. A phased approach to development and deployment is essential. Phase 1 should prioritize robust safety mechanisms and rigorous technical testing of the framework’s individual components in simulated environments. Phase 2 should involve pilot implementations in supervised, blended-care contexts, with early RCTs assessing feasibility, engagement, and preliminary efficacy. Only after robust evidence of safety and effectiveness emerges should the system progress to greater autonomy and advanced personalization (phase 3), and any such expansion must remain under close regulatory and ethical oversight. Advancing along this roadmap will require long-term interdisciplinary collaboration, substantial investment in high-quality data and clinical trials, and the establishment of clear regulatory standards to foster public trust and ensure responsible innovation.

### Conclusion

This paper introduces the MIND-SAFE framework, a comprehensive PE framework, and a conceptual model to guide the development of safer, more effective, and ethically sound LLM-based mental health chatbots. By integrating empirically validated therapeutic models within a layered architecture of technical and clinical safeguards, the framework offers a practical pathway for creating AI-driven tools that deliver empathetic and contextually relevant support.

More than a standalone proposal, this framework is situated within the broader scholarly landscape; its components are explicitly mapped to established evaluative standards, such as the FAITA-MH [25] and READI [26] frameworks, ensuring that development and validation remain consistent with emerging best practices. We move beyond a generic call for evaluation by proposing a pragmatic, ethically grounded validation strategy that compares the full framework against a minimally safe LLM baseline, providing a clear path to generating empirical evidence of its value.

Importantly, this work recognizes that technical safeguards alone are insufficient. We examine the ethical complexities at the intersection of AI and clinical responsibility, including the gap between automated crisis alerts and the legal “duty to protect.” This underscores that the framework is not intended to replace clinicians but to support and augment their work.

Ultimately, translating this model into real-world applications requires a rigorous, phased roadmap shaped by interdisciplinary collaboration and stakeholder engagement. Addressing the ethical dimensions of integrating AI into mental health care, the framework highlights a pressing need: to move beyond algorithmic precision and foster enduring partnerships between clinicians and AI systems, partnerships grounded in therapeutic understanding, guided by clear ethical principles, and validated through continuous, real-world application.

## Abbreviations

ACT: acceptance and commitment therapy
AI: artificial intelligence
APA: American Psychological Association
CBT: cognitive behavioral therapy
DBT: dialectical behavior therapy
FAITA-MH: Framework for AI Tool Assessment in Mental Health
GDPR: General Data Protection Regulation
HIPAA: Health Insurance Portability and Accountability Act
LLM: large language model
MIND-SAFE: Mental well-being through Dialogue – Safeguarded & Adaptive Framework for Ethics
ML: machine learning
PE: prompt engineering
RAG: retrieval-augmented generation
RCT: randomized controlled trial
READI: Readiness Evaluation for AI-Mental Health Deployment and Implementation
USD: user state database

## References

[1] Stade EC, Stirman SW, Ungar LH (2024). Large language models could change the future of behavioral healthcare: a proposal for responsible development and evaluation. Npj Ment Health Res.

[2] Chow JCL, Sanders L, Li K (2023). Impact of ChatGPT on medical chatbots as a disruptive technology. Front Artif Intell.

[3] (2021). Mental health ATLAS 2020. World Health Organization.

[4] (2021). Comprehensive mental health action plan 2013–2030. World Health Organization.

[5] Stein DJ, He Y, Phillips A, Sahakian BJ, Williams J, Patel V (2015). Global mental health and neuroscience: potential synergies. Lancet Psychiatry.

[6] Saxena S, Funk M, Chisholm D (2014). WHO’s Mental Health Action Plan 2013-2020: what can psychiatrists do to facilitate its implementation?. World Psychiatry.

[7] Patel V, Chisholm D, Parikh R (2016). Addressing the burden of mental, neurological, and substance use disorders: key messages from Disease Control Priorities, 3rd edition. Lancet.

[8] Chandrashekar P (2018). Do mental health mobile apps work: evidence and recommendations for designing high-efficacy mental health mobile apps. Mhealth.

[9] Abd-Alrazaq AA, Rababeh A, Alajlani M, Bewick BM, Househ M (2020). Effectiveness and safety of using chatbots to improve mental health: systematic review and meta-analysis. J Med Internet Res.

[10] Torous J, Jän Myrick K, Rauseo-Ricupero N, Firth J (2020). Digital mental health and COVID-19: using technology today to accelerate the curve on access and quality tomorrow. JMIR Ment Health.

[11] Madaan A, Tandon N, Gupta P (2023). Self-refine: iterative refinement with self-feedback. arXiv.

[12] Reynolds L, McDonell K (2024). Prompt programming for large language models: beyond the few-shot paradigm. arXiv.

[13] Meskó B (2023). Prompt engineering as an important emerging skill for medical professionals: tutorial. J Med Internet Res.

[14] Hua Y, Na H, Li Z (2024). Applying and evaluating large language models in mental health care: a scoping review of human-assessed generative tasks. arXiv.

[15] Guo Z, Lai A, Thygesen JH, Farrington J, Keen T, Li K (2024). Large language models for mental health applications: systematic review. JMIR Ment Health.

[16] Marvin G, Hellen N, Jjingo D, Nakatumba-Nabende J, Jacob IJ, Piramuthu S, Falkowski-Gilski P (2024). Data Intelligence and Cognitive Informatics Algorithms for Intelligent Systems.

[17] Lee YK, Lee I, Shin M, Bae S, Hahn S (2023). Chain of empathy: enhancing empathetic response of large language models based on psychotherapy models. arXiv.

[18] Beck JS (2020). Cognitive Behavior Therapy: Basics and Beyond.

[19] Hayes SC, Lillis J (2012). Acceptance and Commitment Therapy: The Process and Practice of Mindful Change.

[20] Linehan MM (1993). Cognitive-Behavioral Treatment of Borderline Personality Disorder.

[21] Sato K, Kaneko H, Fujimura M (2024). Reducing cultural hallucination in non-English languages via prompt engineering for large language models. Open Science Framework.

[22] Belkhir A (2023). Improving ChatGPT’s emotional intelligence through prompt engineering. https://archipel.uqam.ca/17338/1/M18333.pdf.

[23] Kang B, Hong M (2025). Development and evaluation of a mental health chatbot using ChatGPT 4.0: mixed methods user experience study with Korean users. JMIR Med Inform.

[24] Zahran N, Fouda AE, Hanafy RJ, Fouda ME (2025). A comprehensive evaluation of large language models on mental illnesses in arabic context. arXiv.

[25] Golden A, Aboujaoude E (2024). The Framework for AI Tool Assessment in Mental Health (FAITA - Mental Health): a scale for evaluating AI-powered mental health tools. World Psychiatry.

[26] Stade EC, Eichstaedt JC, Kim JP, Stirman SW (2025). Readiness Evaluation for AI-Mental Health Deployment and Implementation (READI): a review and proposed framework. Technol Mind Behav.

[27] Martinengo L, Lin X, Jabir AI (2023). Conversational agents in health care: expert interviews to inform the definition, classification, and conceptual framework. J Med Internet Res.

[28] Owen D, Lynham AJ, Smart SE, Pardiñas AF, Camacho Collados J (2024). AI for analyzing mental health disorders among social media users: quarter-century narrative review of progress and challenges. J Med Internet Res.

[29] Abilkaiyrkyzy A, Laamarti F, Hamdi M, Saddik AE (2024). Dialogue system for early mental illness detection: toward a digital twin solution. IEEE Access.

[30] Karamat A, Imran M, Yaseen MU, Bukhsh R, Aslam S, Ashraf N (2025). A hybrid transformer architecture for multiclass mental illness prediction using social media text. IEEE Access.

[31] Stasolla F, Passaro A, Curcio E (2025). Combined deep and reinforcement learning with gaming to promote healthcare in neurodevelopmental disorders: a new hypothesis. Front Hum Neurosci.

[32] Pralat N, Ischen C, Voorveld H, Følstad A (2025). Chatbots and Human-Centered AI Lecture Notes in Computer Science.

[33] pan siyu, Fan C, Zhao B, Luo S, Jin Y (2024). Can large language models exhibit cognitive and affective empathy as humans?. Open Science Framework.

[34] Pergantis P, Bamicha V, Skianis C, Drigas A (2025). AI chatbots and cognitive control: enhancing executive functions through chatbot interactions: a systematic review. Brain Sci.

[35] Alam L, Mamun TI, Mueller ST (2025). Application of cognitive empathy elements into AI chatbots: an interview study exploring patient-physician interaction. J Cogn Eng Decis Mak.

[36] Liu T, Giorgi S, Aich A (2024). The illusion of empathy: how AI chatbots shape conversation perception. arXiv.

[37] Zhang Y, Yang X, Xu X (2024). Affective computing in the era of large language models: a survey from the NLP perspective. SSRN.

[38] hua yining, Liu F, Yang K (2024). Large language models in mental health care: a systematic scoping review (preprint). JMIR Mental Health.

[39] Abbasian S, Azimi I, Feli M, Rahmani AM (2024). Empathy through multimodality in conversational interfaces. arXiv.

[40] Sorin V, Brin D, Barash Y (2024). Large language models and empathy: systematic review. J Med Internet Res.

[41] Joshi R, Jadeja M (2024). Affective Computing for Social Good: Enhancing Well-Being.

[42] Jain A, Jain A (2025). Generative Artificial Intelligence for Biomedical and Smart Health Care Systems.

[43] Atlam HF, Shafik M, Kurugollu F (2022). Advances in Transdisciplinary Engineering.

[44] Greco D, Barra P, D’Errico L, Staffa M (2024). Multimodal interfaces for emotion recognition: models, challenges and opportunities.

[45] Zhu X, Guo C, Feng H (2024). A review of key technologies for emotion analysis using multimodal information. Cogn Comput.

[46] Zulkarnain R, Hardi R, Syabandyah F AI-powered smart smile: early detection of mental health conditions through computational intelligence.

[47] Chu Y, Liao L, Zhou Z, Ngo CW, Hong R (2024). Towards multimodal emotional support conversation systems. arXiv.

[48] Liu Y, Wang K, Wei L, Chen J, Zhan Y, Tao D (2024). Affective computing for health care: recent trends, applications, challenges, and beyond. arXiv.

[49] Sedlakova J (2024). Ethical and epistemic challenges of conversational AI in mental health care: interdisciplinary inquiry for responsible human–AI interaction [Dissertation]. https://www.zora.uzh.ch/id/eprint/269480/1/269480.pdf.

[50] Manole A, Cârciumaru R, Brînzaș R, Manole F (2025). An exploratory investigation of chatbot applications in anxiety management: a focus on personalized interventions. Information.

[51] Chow JCL, Li K (2025). Developing effective frameworks for large language model-based medical chatbots: insights from radiotherapy education with ChatGPT. JMIR Cancer.

[52] Gkintoni E, Vassilopoulos SP, Nikolaou G (2025). Next-generation cognitive-behavioral therapy for depression: integrating digital tools, teletherapy, and personalization for enhanced mental health outcomes. Medicina (Kaunas).

[53] Li J, Jiang M, Zhao Q, Wang F, He T, Cheng X (2024). A generic review of integrating artificial intelligence in cognitive behavioral therapy. arXiv.

[54] Nelson J, Kaplan J, Simerly G (2025). The balance and integration of artificial intelligence within cognitive behavioral therapy interventions. Curr Psychol.

[55] Katoch H, Jain P, Sharma A, Gautam L, Sharma Y (2025). From algorithms to empathy: navigating ethics, efficacy, and user trust. Int J Interdiscip Approaches Psychol.

[56] Grodniewicz JP, Hohol M (2023). Waiting for a digital therapist: three challenges on the path to psychotherapy delivered by artificial intelligence. Front Psychiatry.

[57] Lawrence HR, Schneider RA, Rubin SB, Matarić MJ, McDuff DJ, Jones Bell M (2024). The opportunities and risks of large language models in mental health. JMIR Ment Health.

[58] Al Foysal A (2024). Chatbots in psychology: revolutionizing clinical support and mental health care. https://www.scirp.org/pdf/vp2024103_92140611.pdf.

[59] Shen H, Li Z, Yang M Are large language models possible to conduct cognitive behavioral therapy?.

[60] Yuan A, Garcia Colato E, Pescosolido B, Song H, Samtani S (2025). Improving workplace well-being in modern organizations: a review of large language model-based mental health chatbots. ACM Trans Manage Inf Syst.

[61] Yu HQ, McGuinness S (2024). An experimental study of integrating fine-tuned large language models and prompts for enhancing mental health support chatbot system. J Med Artif Intell.

[62] Babu A, Joseph AP (2024). Artificial intelligence in mental healthcare: transformative potential vs. the necessity of human interaction. Front Psychol.

[63] Omiyefa S (2025). Artificial intelligence and machine learning in precision mental health diagnostics and predictive treatment models. Int J Res Publ Rev.

[64] Ferrario A, Sedlakova J, Trachsel M (2024). The role of humanization and robustness of large language models in conversational artificial intelligence for individuals with depression: a critical analysis. JMIR Ment Health.

[65] Williams A (2024). Can natural language-based artificial intelligence systems address psychopathology? PCSAS Clinical Psychology Training.

[66] Song I, Pendse SR (2024). The typing cure: experiences with large language model chatbots for mental health support. arXiv.

[67] De Choudhury M, Pendse SR, Kumar N (2023). Benefits and harms of large language models in digital mental health. PsyArXiv.

[68] Held P, Pridgen SA, Chen Y, Akhtar Z, Amin D, Pohorence S (2024). A novel cognitive behavioral therapy-based generative AI tool (Socrates 2.0) to facilitate Socratic dialogue: protocol for a mixed methods feasibility study. JMIR Res Protoc.

[69] Knoth N, Tolzin A, Janson A, Leimeister JM (2024). AI literacy and its implications for prompt engineering strategies. Computers and Education: Artificial Intelligence.

[70] Haque MDR, Rubya S (2023). An overview of chatbot-based mobile mental health apps: insights from app description and user reviews. JMIR Mhealth Uhealth.

[71] Na H (2024). CBT-LLM: a chinese large language model for cognitive behavioral therapy-based mental health question answering. arXiv.

[72] Hu H, Zhou Y, Si J (2025). Beyond empathy: integrating diagnostic and therapeutic reasoning with large language models for mental health counseling. arXiv.

[73] Vitman O, Amaglobeli N, Plachinda P (2024). Dialectical behavior therapy approach to LLM prompting. arXiv.

[74] Omarov B, Zhumanov Z, Gumar A, Kuntunova L (2023). Artificial intelligence enabled mobile chatbot psychologist using AIML and cognitive behavioral therapy. IJACSA.

[75] Martinez-Martin N (2022). Artificial Intelligence in Brain and Mental Health.

[76] Banerjee S, Agarwal A, Bar AK (2024). Securing well-being: exploring security protocols and mitigating risks in AI-driven mental health chatbots for employees. AJCST.

[77] Saxena RR (2024). Applications of natural language processing in the domain of mental health. TechRxiv.

[78] Garcia Valencia OA, Suppadungsuk S, Thongprayoon C (2023). Ethical implications of chatbot utilization in nephrology. J Pers Med.

[79] Bodas H (2024). Optimizing healthcare with AI chatbots: addressing challenges and opportunities. SSRN.

[80] Nasir S, Khan RA, Bai S (2024). Ethical framework for harnessing the power of AI in healthcare and beyond. IEEE Access.

[81] Sharma S, Kaur H, Venkatagiri K, Desai P, Chintala D (2024). Enhancing mental health care with AI: a review discussing biases, methodologies, and clinician preferences. Int J Res Med Sci.

[82] Mennella C, Maniscalco U, De Pietro G, Esposito M (2024). Ethical and regulatory challenges of AI technologies in healthcare: a narrative review. Heliyon.

[83] Li J (2023). Security implications of AI chatbots in health care. J Med Internet Res.

[84] Goktas P, Grzybowski A (2025). Shaping the future of healthcare: ethical clinical challenges and pathways to trustworthy AI. J Clin Med.

[85] Shoghli A, Darvish M, Sadeghian Y (2024). Balancing innovation and privacy: ethical challenges in AI-driven health care. J Rev Med Ethics.

[86] Saeidnia HR, Hashemi Fotami SG, Lund B, Ghiasi N (2025). Ethical considerations in artificial intelligence interventions for mental health and well-being: ensuring responsible implementation and impact. Soc Sci (Basel).

[87] Casu M, Triscari S, Battiato S, Guarnera L, Caponnetto P (2024). AI chatbots for mental health: a scoping review of effectiveness, feasibility, and applications. Appl Sci (Basel).

[88] Boucher EM, Harake NR, Ward HE (2021). Artificially intelligent chatbots in digital mental health interventions: a review. Expert Rev Med Devices.

[89] Manole A, Cârciumaru R, Brînzaș R, Manole F (2024). Harnessing AI in anxiety management: a chatbot-based intervention for personalized mental health support. Information.

[90] Lyons-Cunha J (2024). AI in mental health care: how is it used and what are the risks? Built In.

[91] Li H, Zhang R, Lee YC, Kraut RE, Mohr DC (2023). Systematic review and meta-analysis of AI-based conversational agents for promoting mental health and well-being. NPJ Digit Med.

[92] Choo S, Yoo S, Endo K, Truong B, Son MH (2025). Advancing clinical chatbot validation using AI-powered evaluation with a new 3-bot evaluation system: instrument validation study. JMIR Nurs.

[93] Thomas J, Lucht A, Segler J (2025). An explainable artificial intelligence text classifier for suicidality prediction in youth crisis text line users: development and validation study. JMIR Public Health Surveill.

[94] Cross S, Bell I, Nicholas J (2024). Use of AI in mental health care: community and mental health professionals survey. JMIR Ment Health.

[95] Torous J, Bucci S, Bell IH (2021). The growing field of digital psychiatry: current evidence and the future of apps, social media, chatbots, and virtual reality. World Psychiatry.

[96] Weisenburger RL, Mullarkey MC, Labrada J (2024). Conversational assessment using artificial intelligence is as clinically useful as depression scales and preferred by users. PsyArXiv.

[97] Koutsouleris N, Hauser TU, Skvortsova V, De Choudhury M (2022). From promise to practice: towards the realisation of AI-informed mental health care. The Lancet Digital Health.

[98] Weizenbaum J (1966). ELIZA—a computer program for the study of natural language communication between man and machine. Commun ACM.

[99] Fitzpatrick KK, Darcy A, Vierhile M (2017). Delivering cognitive behavior therapy to young adults with symptoms of depression and anxiety using a fully automated conversational agent (Woebot): a randomized controlled trial. JMIR Ment Health.

[100] Inkster B, Sarda S, Subramanian V (2018). An empathy-driven, conversational artificial intelligence agent (Wysa) for digital mental well-being: real-world data evaluation mixed-methods study. JMIR Mhealth Uhealth.

[101] Chaudhry BM, Debi HR (2024). User perceptions and experiences of an AI-driven conversational agent for mental health support. Mhealth.

[102] Moylan K, Doherty K (2025). Expert and interdisciplinary analysis of AI-driven chatbots for mental health support: mixed methods study. J Med Internet Res.

[103] Fulmer R, Joerin A, Gentile B, Lakerink L, Rauws M (2018). Using psychological artificial intelligence (Tess) to relieve symptoms of depression and anxiety: randomized controlled trial. JMIR Ment Health.

[104] Stephens TN, Joerin A, Rauws M, Werk LN (2019). Feasibility of pediatric obesity and prediabetes treatment support through Tess, the AI behavioral coaching chatbot. Transl Behav Med.

[105] Larson C (2023). Talkspace CEO: AI can improve therapist performance, boost quality. Behavioral Health Business.

[106] Anser MK, Nabi AA, Ahmad I, Abro MMQ, Zaman K (2025). Advancing mental health care: a comprehensive review of digital tools and technologies for enhancing diagnosis, treatment, and wellness. Health Care Sci.

[107] Hanji BR, S C, Gupta D, Krishna E, Devadiga HG Self-heal: conversational therapy bot with AI enhanced features for mental health.

[108] Vineeth R, Maskey S, Vishakan US, Singh Y A proposed chatbot psykh your personal therapist and stress buster using rasa open-source framework.

[109] Mehta A, Niles AN, Vargas JH, Marafon T, Couto DD, Gross JJ (2021). Acceptability and effectiveness of artificial intelligence therapy for anxiety and depression (Youper): longitudinal observational study. J Med Internet Res.

[110] (2021). Major study from stanford university and youper finds artificial intelligence therapy effective at reducing anxiety and depression. PR Newswire.

[111] Balaskas A, Schueller SM, Cox AL, Rashleigh C, Doherty G (2023). Examining young adults daily perspectives on usage of anxiety apps: a user study. PLOS Digit Health.

[112] Bautista J, Liu M, Alvarez M, Schueller SM (2025). Multi-media field test: cognitive-behavioral therapy at our fingertips: Sanvello provides on-demand support for mental health. Cogn Behav Pract.

[113] Garrido S, Cheers D, Boydell K (2019). Young people’s response to six smartphone apps for anxiety and depression: focus group study. JMIR Ment Health.

[114] Sharma G, Schlosser L, Jones BDM (2022). Brief app-based cognitive behavioral therapy for anxiety symptoms in psychiatric inpatients: feasibility randomized controlled trial. JMIR Form Res.

[115] Morse KE, Ostberg NP, Jones VG, Chan AS (2020). Use characteristics and triage acuity of a digital symptom checker in a large integrated health system: population-based descriptive study. J Med Internet Res.

[116] Jungmann SM, Klan T, Kuhn S, Jungmann F (2019). Accuracy of a chatbot (Ada) in the diagnosis of mental disorders: comparative case study with lay and expert users. JMIR Form Res.

[117] Chow JCL, Wong V, Li K (2024). Generative Pre-Trained Transformer-empowered healthcare conversations: current trends, challenges, and future directions in large language model-enabled medical chatbots. BioMedInformatics.

[118] Abrams Z (2025). Using generic AI chatbots for mental health support: a dangerous trend. APA Services.

[119] Dong Y, Mu R, Zhang Y, Sun S, Zhang T, Wu C (2024). Safeguarding large language models: a survey. arXiv.

[120] Paraschiv EA, Băjenaru L, Petrache C, Bica O, Nicolau DN (2024). AI-driven neuro-monitoring: advancing schizophrenia detection and management through deep learning and EEG analysis. Future Internet.

[121] Alhuzali H, Alasmari A (2024). Evaluating the effectiveness of the foundational models for q&a classification in mental health care. arXiv.

[122] (2024). Ethical considerations in AI chatbot design. AgentX.

[123] Noble JM, Zamani A, Gharaat M (2022). Developing, implementing, and evaluating an artificial intelligence-guided mental health resource navigation chatbot for health care workers and their families during and following the COVID-19 pandemic: protocol for a cross-sectional study. JMIR Res Protoc.

[124] Chametka P, Maqsood S, Chiasson S Security and privacy perceptions of mental health chatbots.

[125] Balcombe L (2023). AI chatbots in digital mental health. Informatics (MDPI).

[126] Xue J, Wang YC, Wei C, Liu X, Woo J, Kuo CCJ (2023). Bias and fairness in chatbots: an overview. arXiv.

[127] Waaler PN, Hussain M, Molchanov I, Bongo LA (2024). Prompt engineering a schizophrenia chatbot: utilizing a multi-agent approach for enhanced compliance with prompt instructions. arXiv.

[128] Truong L, Lee S, Sawhney N (2024). Enhancing conversations in migrant counseling services: designing for trustworthy human-AI collaboration. Proc ACM Hum-Comput Interact.

[129] Aleem M, Zahoor I, Naseem M Towards culturally adaptive large language models in mental health: using ChatGPT as a case study.

[130] Wang X, Sanders HM, Liu Y (2023). ChatGPT: promise and challenges for deployment in low- and middle-income countries. The Lancet Regional Health - Western Pacific.

[131] Chow JCL, Li K (2024). Ethical considerations in human-centered AI: advancing oncology chatbots through large language models. JMIR Bioinform Biotechnol.

[132] Anisha SA, Sen A, Bain C (2024). Evaluating the potential and pitfalls of AI-powered conversational agents as humanlike virtual health carers in the remote management of noncommunicable diseases: scoping review. J Med Internet Res.

[133] Liu I, Liu F, Xiao Y, Huang Y, Wu S, Ni S (2025). Investigating the key success factors of chatbot-based positive psychology intervention with retrieval- and Generative Pre-Trained Transformer (GPT)-based chatbots. International Journal of Human–Computer Interaction.

[134] Martinengo L, Lum E, Car J (2022). Evaluation of chatbot-delivered interventions for self-management of depression: content analysis. J Affect Disord.

[135] Vossen W, Szymanski M, Verbert K The effect of personalizing a psychotherapy conversational agent on therapeutic bond and usage intentions.

[136] Ulrich S, Lienhard N, Künzli H, Kowatsch T (2024). A chatbot-delivered stress management coaching for students (MISHA app): pilot randomized controlled trial. JMIR Mhealth Uhealth.

[137] (2020). G-3: guidance on research activities not requiring IRB approval. Grand Valley State University.

[138] Appelbaum PS (1985). Tarasoff and the clinician: problems in fulfilling the duty to protect. Am J Psychiatry.

[139] Bloom JD, Rogers JL (1988). The duty to protect others from your patients--Tarasoff spreads to the Northwest. West J Med.

[140] Doraiswamy PM, Blease C, Bodner K (2020). Artificial intelligence and the future of psychiatry: Insights from a global physician survey. Artif Intell Med.

[141] Miner AS, Milstein A, Schueller S, Hegde R, Mangurian C, Linos E (2016). Smartphone-based conversational agents and responses to questions about mental health, interpersonal violence, and physical health. JAMA Intern Med.

[142] Luxton DD (2014). Recommendations for the ethical use and design of artificial intelligent care providers. Artif Intell Med.

